# Comprehensive Genome-Wide Analysis of Dmrt Transcription Factors Reveals Their Roles in Sexual Dimorphism in *Scolopendra mutilans*

**DOI:** 10.3390/biology14101451

**Published:** 2025-10-20

**Authors:** Zhiqiang Li, Jingjing Guo, Ghulam Nabi, Zheng Wang, Buddhi Dayananda, Lin Zhang

**Affiliations:** 1 Hubei Shizhen Laboratory, Key Laboratory of Chinese Medicinal Resource and Chinese Herbal Compound of Ministry of Education, School of Basic Medical Sciences, Hubei University of Chinese Medicine, Wuhan 430065, China; lizhiqianghihihi@outlook.com (Z.L.); aliaoyx@163.com (J.G.); 2 Center for Animal Sciences and Fisheries, The University of Swat, Khyber Pakhtunkhwa, Swat 19200, Pakistan; ghulamnabiqau@gmail.com; 3 College of Life Sciences, Nanjing Forestry University, Nanjing 210037, China; zhengwang@njfu.edu.cn; 4 School of Agriculture and Food Sciences, The University of Queensland, Brisbane, QLD 4072, Australia; b.dayananda@uq.edu.au

**Keywords:** Dmrt transcription factors, gene expression, *Scolopendra mutilans*, sexual determination

## Abstract

**Simple Summary:**

In this study, we identified eight Dmrt transcription factors in the genome of the centipede *Scolopendra mutilans*. Phylogenetic analysis revealed evolutionary conservation across arthropods, and structural analysis confirmed conserved DM domains and sex-specific motifs, with tan-dem duplication of Dsx2 on chromosome 4. Correlation analysis linked the expression of *Sxl* and *Fem-1C* to the regulation of *Dsx* isoforms, suggesting the presence of a conserved upstream regulatory cascade for sex-specific splicing.

**Abstract:**

The double-sex and mab-3-related transcription factors (Dmrt) are widely distributed in the animal kingdom and play a crucial role in sex determination and differentiation. In this study, we identified eight Dmrt transcription factors in the genome of the centipede *Scolopendra mutilans*, including five *Dsx*-related genes (as *Dsx1*, *Dsx2* (five splice variants), *Dsx3*, *Dsx4* and *Dsx5*) and three *Dmrt*-related genes (as *Dmrt11E*, *Dmrt99B*, and *Dmrt93B*). Phylogenetic analysis revealed evolutionary conservation across arthropods, with *Dsx* genes clustered into class-specific clades (Chilopoda, Insecta, Crustacea, Arachnida). Structural analysis confirmed conserved DM domains and sex-specific motifs, with tandem duplication of *Dsx2* on chromosome 4. Expression profiling demonstrated significant sexual dimorphism: *Dsx5* was female-biased, whereas *Dsx2*, *Dsx3*, and *Dsx4* were male-biased, suggesting their functional divergence in sexual differentiation. Correlation analysis linked the expression of *Sxl* and *Fem-1C* to the regulation of *Dsx* isoforms, suggesting the presence of a conserved upstream regulatory cascade for sex-specific splicing. These findings elucidate the structural and functional landscape of *Dmrt*s in *S. mutilans*, and provide insights into how sex-determination mechanisms evolved in Myriapoda.

## 1. Introduction

Sexual reproduction is a fundamental phenomenon across the animal kingdom. During reproduction, sex determination is the result of a complex mechanisms primarily regulated by genetic sex determination (GSD), environmental sex determination (ESD), or a combination of both [1]. In ESD species, the environmental factors such as temperature determine the sex during embryonic development [2]. In contrast, GSD species rely on a master sex-determining gene to direct gonadal development into either testes or ovaries [3]. Recent studies have led to the discovery of multiple genes implicated in sexual development, such as double-sex and mab-3-related transcription factor (*Dmrt*), feminizer (*Fem-1*), Forkhead box (*Fox*), SRY-related HMG-box (*Sox*), signal transducer and activator of the transcription factor (*STAT*), Sex-lethal (*Sxl*), transforming growth factor-β family genes (*TGF-β*), and wingless-type mmtv integration site family (*Wnt*). Many of these genes demonstrate sexually dimorphic expression patterns within gonadal tissue, underscoring their essential functions in sex determination, gonadal differentiation, and maintenance [4]. In vertebrates, sex differentiation is governed by the female pathway (*Rspo1*/*Wnt*/*β-catenin/Foxl2*) and male pathway (*Sry*/*Sox9*/*Fgf9/Dmrt1*) [5]. In Eumetazoa, *Dmrt* are evolutionarily conserved components of the sex determination cascade. In Nematoda and Arthropoda, *Fem-1* has been functionally linked to regulatory pathways involving *Dmrt* homologs. In insects, *tra* (transformer) has been described as the critical regulator of *Dmrt* sex-specific splicing [6]. In *Drosophila melanogaster*, the presence of Sxl protein activates *tra*, generating *DsxF*, which then produces female traits. In contrast, the absence of Sxl protein generates *DsxM,* producing male traits [7,8,9].

Dmrt (doublesex and mab-3-related) proteins are a class of transcription factor encoded by homologous genes with *Doublesex* (*Dsx*) in *D. melanogaster* and *mab-3* in *Caenorhabditis elegans*. These proteins are characterized by a conserved double-sex and mab-3 domain, and were first discovered in *D. melanogaster* [10]. *Dmrt* are considered master regulators of sex determination and sexual differentiation across the animal kingdom [11,12,13]. The number of *Dmrt* genes varies across among taxon. In humans, there are eight members (*Dmrt1*–*Dmrt8*) [14], in mice, seven (*Dmrt1*–*Dmrt7*) [15], and in fish, seven as well (*Dmrt1–Dmrt6* plus the paralog *Dmrt2b*) [16]. Notably, two mammalian-specific *Dmrt* genes have been identified and designated as *Dmrt7* and *Dmrt8* [17], highlighting the dynamic evolution of this transcription factor. Phylogenetic analyses of the *Dmrt* have revealed strongly conserved orthologous groups spanning diverse metazoan lineages. The mammalian *Dmrt2/2a* [18,19], originally identified in human [20], shares homologous relationships with fish *Dmrt2b/terra* [21,22], bivalve *Dmrt* [23], and arthropod *Dmrt11E* [24]; these orthologous were clustered into the Dmrt2 group. Similarly, the vertebrate *Dmrt4/5* [25], arthropod *Dmrt99B* [26], and nematode *Dmd5* [27] were grouped into the Dmrt4/5 group. Interestingly, the invertebrate *Dmrt93B* [28], vertebrate *Dmrt3* [29], and nematode *Dmd4* [30] were clustered into the Dmrt93B group. The high conservation of these clusters suggests strong selective pressure to preserve their developmental and reproductive functions [13]. Compared to invertebrates, the members of *Dmrt* as *Dmrt1*, *Dmrt7* and *Dmrt8* are unique to vertebrates [24], whereas *Dsx* is only restricted to arthropods [31]. The number of *Dsx* genes varies significantly among arthropod species.

In the teleost fish *Oryzias latipes*, the Y chromosome-linked gene *Dmrt1bY* (also known as *DMY*), a paralogue of *Dmrt1*, functions as the primary sex-determining factor governing male differentiation, fulfilling a role functionally analogous to that of mammalian *Sry* [32]. In *Xenopus laevis*, *DMW*, the W chromosome-linked paralogue of *Dmrt1*, acts as a dominant-negative inhibitor of masculinization, suppressing testis differentiation and promoting ovarian development [33]. In pan-arthropod, Dmrt genes are clustered into four groups, designated as *Dsx*, *Dmrt11E*, *Dmrt93B* and *Dmrt99B* [29]. *Dsx-like* is the *Dsx* homolog identified in *Eriocheir sinensis* [34]. The absence of the DMA domain in a gene, despite its homology to *Drosophila Dmrt99B* and vertebrate *Dmrt5*, led to its designation as a *Dmrt-like* gene [35]. *iDMY* is the Y-linked *Dmrt* gene identified in *Sagmariasus verreauxi* [36].

*Scolopendra mutilans* Koch (Scolopendromorpha: scolopendridae) is widely distributed across China [37], and represents the most common and largest centipede species in the middle and lower reaches of the Yangtze River. It plays a crucial role in stabilizing the local soil ecosystem [38]. As a medically important invertebrate in China, most studies on *S. mutilans* have focused on its captive breeding and medicinal properties, including peptide toxin [39,40,41]. Notably, *S. mutilans* exhibits significant sexual dimorphism (unpublished data). Females generally possess a larger body size (including body length and body width) and display faster growth rates compared to males. Venom production correlates positively with body size in *S. subspinipes* and *S. polymorpha* [42], suggesting that female *S. mutilans* likely holds greater economic and medicinal value.

Beyond their canonical role in sex determination and differentiation, the pleiotropic functions of the evolutionarily conserved *Dmrt* genes encompass essential roles in embryonic development across invertebrates and vertebrates. These genes regulate a wide range of biological processes during embryo development and sexual differentiation. We hypothesized that differential expression of *Dmrt* is associated with sexual dimorphism in *S. mutilans*, potentially leading to transcriptional differences between adult males and females. To test this, we conducted a genome-wide analysis to identify the Dmrt transcription factors in *S. mutilans* and examined their physicochemical properties, structure features, and phylogenetic relationships. Additionally, we compared the expression profiles of Dmrt transcription factors between the two sexes. This study provides a genomic framework for understanding Dmrt transcription factor in *S. mutilans* and establishes a foundation for further research on sexual differentiation mechanisms in Myriapoda.

## 2. Materials and Methods

### 2.1. Identification and Characterization of Dmrt Transcription Factor in S. mutilans

To identify the potential Dmrt transcription factor members in *S. mutilans*, we employed the advanced HMMER search (version 3.2.1) using the Hidden Markov Model (HMM) of Dmrt (PF00751) and Basic Local Alignment Search Tool for Proteins (BLASTp) analysis with published Dmrt protein sequences from *Bombyx mori*, *D. melanogaster*, *Daphnia magna*, *E. sinensis*, *Euperipatoides kanangrensis*, *Glomeris marginata*, *Procambarus clarkii*, *Tribolium castaneum*, and *X. laevis* as query sequences (with e-value ≤ 1 × 10^−5^). These analyses were conducted in TBtools v2.142 [43] based on the local *S. mutilans* genome database [44]. These candidates’ Dmrt transcription factors were further validated by screening conserved domains and motifs using NCBI’s Conserved Domain Database (https://www.ncbi.nlm.nih.gov/cdd, accessed on 23 December 2024), the Simple Modular Architecture Research Tool (http://smart.embl-heidelberg.de/, accessed on 26 December 2024) and the Multiple EM for Motif Elicitation suite (http://meme-suite.org/tools/meme, accessed on 24 December 2024). Subsequently, those conversed domains and motifs were visualized using the Gene Structure View (Advanced) and multiple amino acid sequence alignments functions in TBtools (version 2.357).

To gain insight into the conservation and variation in the DM domain across different species and groups including Dmrt1, Dmrt99B, Dmrt93B, Dmrt11E, Dsx-Malacostraca and Dsx-non-Malacostraca from different species, three-dimensional structures of the DM domains were generated by homology modeling employing SWISS-MODEL server (https://swissmodel.expasy.org/, accessed on 16 Match 2025). The structural alignment and the root mean square deviation (RMSD) calculations were carried out with PyMOL (version 3.1), and all protein molecules’ 3D structural figures were visualized using the same software.

In addition, we determined the several physicochemical properties of all Dmrt transcription factor proteins, including amino acid sequence lengths, molecular weight (MW), isoelectric point (pI), and grand average of hydropathy, employing ProtParam (https://web.expasy.org/protparam/, accessed on 27 December 2024) in TBtools (version 2.357). Then, we employed ProtComp 9.0 (http://www.softberry.com/, accessed on 27 December 2024) to predict the subcellular location of all Dmrt transcription factors.

### 2.2. Molecular Phylogenetic Analysis

A phylogenetic tree was reconstructed using the Dmrt transcription factor sequences from *S. mutilans* and others 37 other species, including *D. melanogaster*, *D. magna*, and *G. marginata* (for details, see Supplementary Materials). L-INS-Ioption (the most accurate model) was selected and the MAFFT software (version 7.526) was employed to perform the multiple-sequence alignment and manually trimmed. Then, the maximum-likelihood (ML) phylogenetic tree was conducted employing the IQ-TREE software (version 2.40) with 1000 ultrafast bootstrap replicates to assess branch support [45]. The best-fit substitution model (VT+F+R5) was automatically selected using the “-MPF” option implemented in IQ-TREE. Finally, phylogenetic consensus tree visualization and annotation were carried out in iTOL version 7.2.2 (https://itol.embl.de, accessed on 27 December 2024) and further refined using the *itol.toolkit* package (version 1.1.7) in R (version 4.3.1) [46].

### 2.3. Chromosome Locations and Synteny Analysis

The chromosomal location information of the *Dmrt* transcription factors was derived from the genomic annotation data of *S. mutilans*. The visualization of the chromosomal distribution pattern of the Dmrt transcription factors was accomplished using TBtools. MCSanX (version 1.0.0, the minimum match size as 5, max gap-size as 25, gap penalty as −1, e-value as 1 × 10^−10^) was employed to carry out collinearity analyses of all *Dmrt* transcription factors across 11 species genomes, including *Apis mellifera*, *Cordylochernes scorpioides*, *D. melanogaster*, *Parasteatoda tepidariorum*, *Penaeus vannamei*, *Periplaneta americana*, *Portunus trituberculatus*, *P. clarkii*, *Thereuonema tuberculata, T. castaneum*, and *S. mutilans* [47].

### 2.4. The PPI Networks of Dmrt Transcription Factors

To generate the protein–protein interaction (PPI) network, we submitted the 12 *Dmrt* transcription factor sequences to the STRING database (version 12.0, https://string-db.org/ accessed on 21 March 2025), selecting *D. melanogaster* as the reference organism. Subsequently, functional enrichment analysis was performed within STRING 12.0 to identify the gene ontology (GO) and Kyoto Encyclopedia of Genes and Genomes (KEGG) pathway by default parameters.

### 2.5. The Expression Pattern of Dmrt Transcription Factors in Males and Females

Expression profiles of the Dmrt transcription factors were analyzed using RNA-seq data derived from six male and six female individuals. All reads were aligned to the *S. mutilans* genome, and gene expression levels were quantified as FPKM values using Cufflinks (version 2.2.1, http://cole-trapnell-lab.github.io/cufflinks, accessed on 12 April 2025). To identify sex-biased expression, we compared transcript levels between the male and female groups. The expression patterns were visualized in the form of a heatmap generated with the *pheatmap* package (version 1.0.12) in R (version 4.3.1), based on *Z*-score normalized values across genes.

## 3. Results

### 3.1. Structure and Physicochemical Traits of the Dmrt Transcription Factors

**Figure 1:**
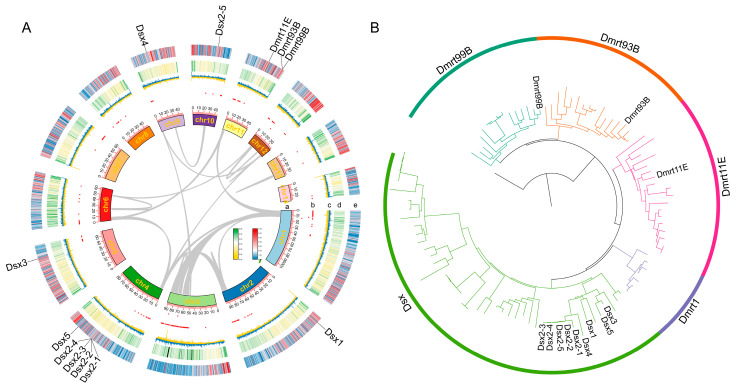
The chromosome distribution (**A**) of Dmrt transcription factors in *Scolopendra mutilans*, and the phylogenetic tree of Dmrt transcription factors in arthropods (**B**) a, chromosome skeleton; b, N ratio; c, GC skew; d, GC ratio; e, gene density.

**Figure 2:**
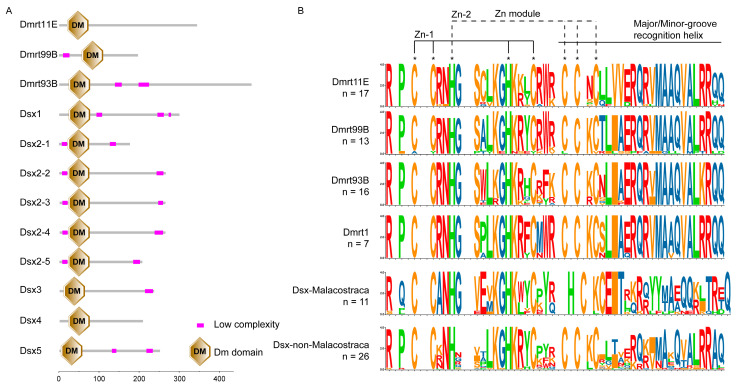
The conserved domain of Dmrt transcription factors in *Scolopendra mutilans* (**A**), and the consensus analysis of DM domains of the Dmrt transcription factors in arthropods (**B**). Less conserved points have been discarded. The size of n is the number of species involved. Amino acids corresponding to Zn^2+^ binding sites are marked with *.

In this present study, 12 putative proteins of the Dmrt transcription factors were identified in *S. mutilans*, including Dmrt11E, Dmrt93B, Dmrt99B, Dsx1, Dsx2 (containing Dsx2-1, Dsx2-2, Dsx2-3, Dsx2-4 and Dsx2-5), Dsx3, Dsx4 and Dsx5 (Figure 1A). All Dmrt transcription factors contained a DM structural domain and a different number of low complexity (LC), such as Dsx1 with 3 LCs, Dsx2 and Dsx5 with 2 LCs (but the location of LCs in Dsxe5 was different from Dsx2), Dsx3 with one LC, and no LCs in Dsx4 (Figure 2A).

The DMRT DM motif was a cysteine-rich DNA-binding domain that included interwoven CCHC and HCCC Zn2+ binding sites, as well as a putative nNLS composed of KGHKK/R (Figure 2B). Protein motif analysis showed that Motif1 and Motif2 existed in all proteins, Motif6 existed only in Dmrt93B, Motif5 and Motif4 existed only in Dmrt11E, Motif7 existed only in Dsx, and Motif9 existed only in Dsx2 and Dsx5 (Figure S1). The 3D structure of the Dmrts in *S. mutilans* DM domains was found to be highly consistent in spatial folding (Figure S2). The RMSD values among these aligned structures vary between 0.007 and 0.041 (Figures S2 and S3), further supporting the high degree of structural conservation within the Dmrt protein family throughout evolution.

The number of amino acids encoded by the Dmrt ranged from 177 (Dsx2 to 1) to 480 (Dmrt93B), with molecular weights ranging from 19.77 (Dsx2-1) to 52.59 (Dmrt93B) kDa, and the predicted PI ranging from 7.55 (Dsx2-4) to 9.69 (Dmrt11E) (Table 1). All Dmrt members exhibited high hydrophilicity in *S. mutilans*. These proteins were predicted to localize in the nucleus, except for Dsx5 and Dsx3, which were predicted to be membrane-bound extracellular locations (Table 1).

### 3.2. Chromosome Locations and Synteny

**Figure 3:**
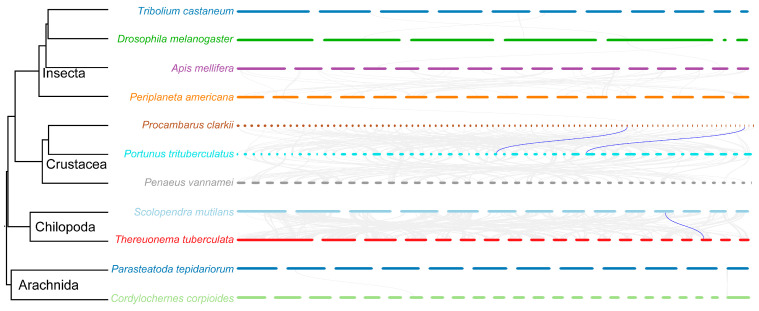
Synteny analysis of Dmrt transcription factors among the genome of arthrpods. The gray lines represent all the syntenic gene pairs, and the blue lines represent the syntenic Dmrt transcription factors gene pairs.

The *Dmrt* transcription factors were distributed across chromosomes (Figure 1A), including Dmrt11E, Dmrt99B and Dmrt93B on chromosome 11, but Dsx on different chromosomes, such as Dsx1 on chromosome 2, Dsx2 (expect Dsx2-5 on chromosome 9) and Dsx5 on chromosome 4, Dsx3 on chromosome 5, and Dsx4 on chromosome 10. We employed synteny analysis among 11 arthropods. However, for Dmrt transcription factors, there were only *Dmrt11E* gene pairs between *T. tuberculata* and *S. mutilans*, with a *Dmrt93B* gene pair between *P. clarkii* and *P. trituberculatus* (Figure 3). Furthermore, there were no gene pairs within *S. mutilans*, and tandem duplications only occurred in *Dsx2*, except for *Dsx2-5* in *S. mutilans* (Figure 1A).

### 3.3. Phylogenetic Analysis

To elucidate the phylogenetic relationships among the Dmrt family, we reconstructed a phylogenetic tree using the ML method (Figure 1B). Dmrt99B and Dmrt93B formed a clade whereas Dsx and Dmrt1 formed another clade, which was subsequently associated with Dmrt11E to form a sister clade. The Dsx clade was further divided into four subclades, including the Arachnida Dsx clade, Insecta Dsx clade, Chilopoda Dsx clade, and Crustacea Dsx clade. *S. mutilans* and *G. marginata* were clustered with *T. tuberculata* in Dmrt99B. Dmrt93B from *G. marginata*, *E. kanangrensis*, *P. chinensis*, *S. mutilans* and *T. tuberculata* formed a subclade. In the Dmrt11e clade, centipedes, including *S. mutilans* and *T. tuberculata*, were grouped together, highlighting lineage-specific conservation.

### 3.4. The PPI Networks of Dmrt Transcription Factors

**Figure 4:**
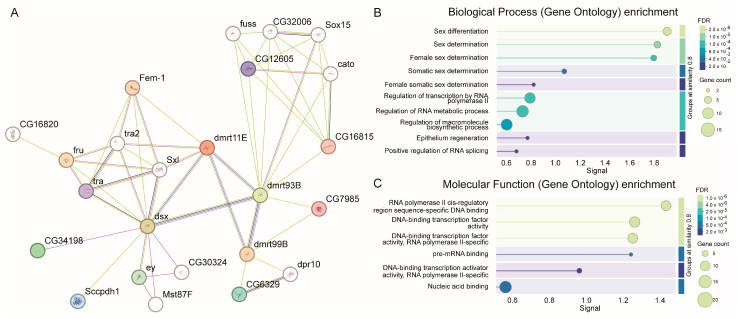
The PPI networks (**A**), biological process (**B**) and molecular function (**C**) of Dmrt transcription factors in *Scolopendra mutilans*.

We examined 12 putative Dmrt transcription factor sequences in the STRING database and identified 12 matched proteins, which were classified into four protein groups. The PPI network comprised 24 nodes and 54 edges with an average node degree of 4.5, and an ap-value of PPI enrichment of 5.96 × 10^−10^ (Figure 4A). GO enrichment analysis indicated that these proteins were primarily associated with biological processes such as sex differentiation (GO:0007548), sex determination (GO:0007530), female sex determination (GO:0030237), somatic sex determination (GO:0018993) and female somatic sex determination (GO:0019101) (Figure 4B). For molecular function, the proteins were enriched in DNA-binding transcription activator activity, RNA polymerase II-specific (GO:0001228), RNA polymerase II cis-regulatory region sequence-specific DNA binding (GO:0000978), DNA-binding transcription factor activity (GO:0003700), DNA-binding transcription factor activity, RNA polymerase II-specific (GO:0000981), pre-mRNA binding (GO:0036002), and nucleic acid binding (GO:0003676) for molecular function (Figure 4C). Additionally, the male semi-fertile phenotype (FBcv:0000006) in *Drosophila* is associated with Dmrt11E and Dsx, highlight the functional relevance of these transcription factors in sex determination.

### 3.5. Expression Profile of Dmrt Transcription Factors in Individuals of Both Sexes

**Figure 5:**
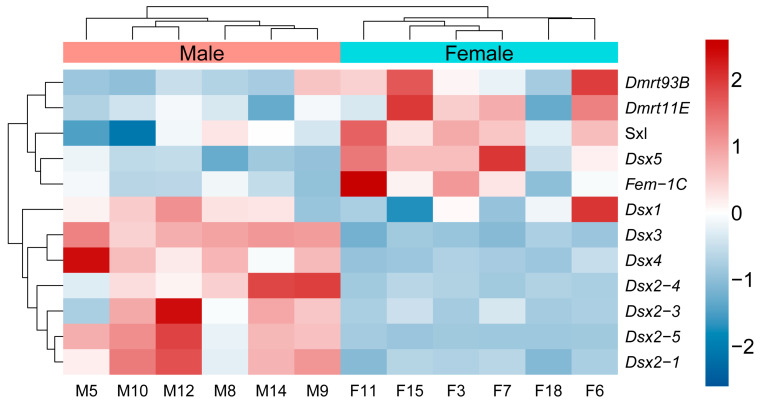
The expression heatmap of Dmrt transcription factors in *Scolopendra mutilans*.

**Figure 6:**
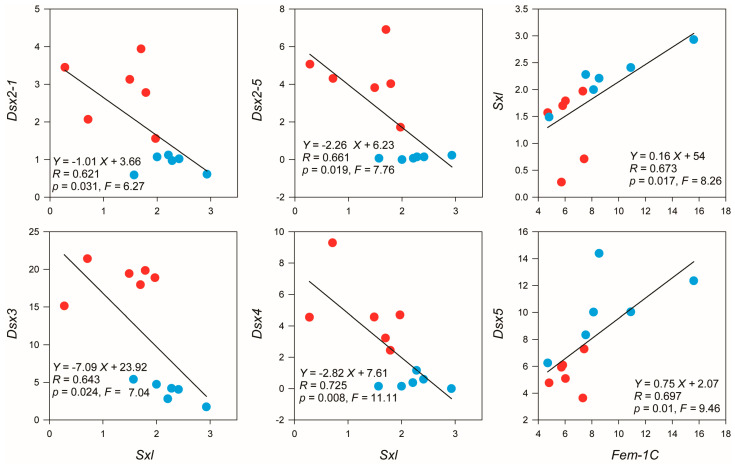
Relationship between *Sxl* and *Dsx*, between *Fem-1C* and *Dsx5*, and between *Fem-1C* and *Sxl* in *Scolopendra mutilans*. Red: male individuals; bule: female individuals.

*Dsx99B* in *S. milantus* demonstrated no expression (PFKM = 0). However, *Dsx5* exhibited a substantially higher expression level in females compared to males (*p* < 0.01, LogFC = −0.90), while *Dsx2-1* (*p* < 0.01, LogFC = 1.65), *Dsx2-3* (*p* < 0.05, LogFC = 3.30), *Dsx2-4* (*p* < 0.01, LogFC = 4.14), *Dsx2-5* (*p* < 0.001, LogFC = 5.31), *Dsx3* (*p* < 0.001, LogFC = 2.30), and *Dsx4* (*p* < 0.001, LogFC = 3.55) showed a pronounced increase in expression levels in males relative to females (Figure 5). The expression level indicated a negative correlation between *sxl* and *Dsx2-1* (*F* = 6.265, *R* = 0.621, *p* < 0.05; Figure 6), between *sxl* and *Dsx2-5* (F = 7.757, *R* = 0.661, *p* < 0.05; Figure 6), between *Sxl* and Dsx3 (*F*_1,10_ = 7.039, *R* = 0.643, *p* < 0.05; Figure 6), and between *sxl* and *Dsx4* (*F*_1,10_ = 11.11, *R* = 0.725, *p* < 0.01; Figure 6). The expression of *Fem-1C* and *Dsx5* was positively related (*F*_1,10_ = 9.462, *R* = 0.697, *p* < 0.05; Figure 6), and a similar trend was reported between the expression of *Fem-1C* and *Sxl* (*F*_1,10_ = 8.261, *R* = 0.673, *p* < 0.05; Figure 6).

## 4. Discussion

We identified eight members among the Dmrt transcription factors members in *S. mutilans* (Figure 1A), located on six chromosomes, including a remarkable tandem duplicate of the *Dsx2* gene (*Dsx2-1* to *Dsx2-5*) on chromosome 4, except for *Dsx2-5*. We reported the *Dmrt11E* gene pair between *T. tuberculata* and *S. mutilans*; however, no gene pairs were found within *S. mutilans*. The expression analysis uncovered sexually dimorphic patterns for several key genes: *Dmrt11E* was female-biased, while multiple *Dsx* isoforms exhibited either male- (*Dsx2*, *Dsx3*, *Dsx4*) or female-biased (*Dsx5*) expression.

The *Dmrt93B* cluster includes orthologs such as vertebrate *Dmrt3* [29] and *Dmd-4* [30]. *Dmrt3* is typically associated with testicular development and embryogenesis [16,48,49,50,51,52]. However, in *S. mutilans*, the Dmrt93B transcript levels were strikingly low in both sexes, suggesting a divergent regulatory role compared to other species. Sexual dimorphism is also observed in the *Dmrt99B* gene. While it is testis-biased in *Scylla paramamosain* and *Macrobrachium rosenbergii*, consistent with the typical role of *Dmrt99B* genes in male development [26,53], it is female-specific in *Daphnia magna* and *Bombyx mori* [54,55]. Intriguingly, *Dmrt99B* was undetectable in *S. mutilans* transcriptome data, raising questions about whether this reflects species-specific gene loss or functional replacement by another gene, which warrants further investigation.

*Dmrt11E* is an important gene in gonadal maturation; however, the expression profile of the *Dmrt11E* gene is complex. The function of Dmrt11E is similar to Dmrt2 [24], playing a key role in the male reproductive physiology of many species. In *S. mutilans*, *Dmrt11E* was highly expressed in females compared to males, a pattern similar to *D. magna* [56] but opposite to that observed in *M. rosenbergii* [26] and *M. nipponense* [57]. This striking divergence suggests that the functional role of *Dmrt11E* is not conserved across the arthropod phylogeny and may have been co-opted for female-specific functions in certain lineages, including myriapods and branchiopods.

*Dsx* undergoes sex-specific alternative splicing to produce male (*DsxM*) and female (*DsxF*) isoforms, which play a crucial role in controlling sexual dimorphism. While this core mechanism is conserved, its regulation and complexity exhibit remarkable lineage-specific variation. The number of alternative transcripts differs among insects; the default splicing pattern in the absence of regulators can be male in *D. melanogaster* or female in *Antheraea mylitta* and *Bombyx mori* [58,59,60,61]. Insect and crustacean studies suggest that upstream regulators like *Sxl* and *Tra-2* may also interact with *Dmrt* genes in a species-specific manner [62,63,64,65,66,67]. Given the evolutionary context, where the core function of Dsx is conserved but its regulatory inputs and molecular details are flexible, we hypothesized that *S. mutilans* similarly utilizes sex-specific *Dsx* isoforms. The results supported this hypothesis, leading us to designate the male-biased isoforms as *SmDsxM* and the female-biased *Dsx5* as *SmDsxF*.

In *D. magna*, *Dsx1* is essential for the development of male-specific traits [68]. However, *Dsx* expression varies significantly among species. In *Cherax quadricarinatus* [69] and *S. paramamosain* [70], *Dsx* expression is significantly higher in the ovary than in the testis. Conversely, in *Penaeus monodon*, *Dsx* expression is significantly lower in the ovary [13]. Our results revealed that *Dsx* expression levels were generally higher in males than in females in *S. mutilans*, except for *Dsx5* (Figure 5). These results suggest that *Dsx* may be functionally divergent, with some isoforms promoting male-specific traits and others regulating female-specific development.

In *S. mutilans*, we observed a sexually dimorphic expression pattern of *Sxl* and *Fem-1C*, with significantly higher expression levels in females than in males. Correlation analysis revealed significant associations between *Sxl* and *Dsx*, *Fem-1C* and *Dsx*, and *Sxl* and *Fem-1C* (Figure 6), suggesting a potential functional relationship among these sex determination genes. Notably, the expression pattern of *Fem-1C* in *S. mutilans* resembles that reported in *Dendroctonus armandi* [71]. The molecular mechanism of sex determination involving these genes appears to follow a conserved regulatory cascade, where the spliced form of the Fem protein regulates the alternative splicing of *dsx* transcripts, while the un-spliced *Fem* remains non-functional and fails to process *Dsx* [72]. This splicing cascade is crucial for proper sexual differentiation in honey bees [73]. Based on the expression profiling, we classified *Dsx2*, *Dsx3,* and *Dsx4* as male-biased *Dsx* isoforms (renamed as *SmDsxM*), while *Dsx5* was identified as a female-biased isoform (renamed as *SmDsxF*). The results suggest that *Fem-1C* may play a dual regulatory role in *S. mutilans*, potentially up-regulating *SmDsxF* expression while down-regulating *SmDsxM*, similar to the regulatory mechanisms observed in other arthropods.

## 5. Conclusions

In this study, we investigated the Dmrt transcription factors in *S. mutilans* and examined their potential roles in sexual determination and differentiation. We identified eight *Dmrt* genes (*Dsx1*, *Dsx2* (with five different alternative splices), *Dsx3*, *Dsx4*, *Dsx5*, *Dmrt11E*, *Dmrt99B,* and *Dmrt93B*). Based on their sexually dimorphic expression patterns, we proposed a sex-specific regulatory model. Our results reveal that terminal *Dsx* signal itself can be complex, the results supporting a model of sexual differentiation driven by the balance between *SmDsxM* and *SmDsxF*. *Dsx5* (renamed *SmDsxF*) was significantly upregulated in females. In contrast, *Dsx2*, *Dsx3*, and *Dsx4* (collectively renamed as *SmDsxM*) exhibited male-biased expression. These findings provide a genomic framework for future functional analyses aimed at understanding *Dmrt*-mediated sexual development in *S. mutilans*, and highlight both conserved and divergent mechanisms within arthropods.

## Figures and Tables

**Table 1:** Physicochemical properties of Dmrt transcription factors in *Scolopendra mutilans*.

ID	Number of Amino Acids	Molecular Weight (Da)	Theoretical pI	Instability Index	Aliphatic Index	GRAVY	Subcellular Location
Dsx1	300	32,302.57	8.91	66.05	76.43	−0.293	Nuclear
Dsx2-1	177	19,774.36	9.68	69.67	59.55	−0.907	Nuclear
Dsx2-2	266	29,851.83	8.36	53.97	77.03	−0.445	Nuclear
Dsx2-3	264	29,788.82	8.39	52.77	77.23	−0.455	Nuclear
Dsx2-4	264	29,751.75	7.55	54.6	78.33	−0.445	Nuclear
Dsx2-5	207	22,860.77	8.6	63.18	69.76	−0.569	Nuclear
Dsx3	236	26,579.16	8.9	56.37	75.68	−0.511	Membrane bound Extracellular
Dsx4	208	23,368.56	9.21	48.73	64.28	−0.562	Nuclear
Dsx5	251	28,179.16	9.15	60.78	83.19	−0.36	Membrane bound Extracellular
Dmrt11E	344	38,643.05	9.69	60.01	64.07	−0.68	Nuclear
Dmrt93B	480	52,594.29	8.11	61.38	65.1	−0.58	Nuclear
Dmrt99B	197	20,602.17	9.02	44.41	68.12	−0.39	Nuclear

GRAVY: grand average of hydropathicity.

## Data Availability

Publicly available datasets were analyzed in this study. This data can be found for *Scolopendra mutilans* (PRJCA021141/CRA030653) at https://ngdc.cncb.ac.cn/gsa/browse/CRA030653.

## Supplementary Materials

The following supporting information can be downloaded at https://www.mdpi.com/article/10.3390/biology14101451/s1: Figure S1: Protein motif analysis of Dmrt transcription factors; Figure S2: Structural alignment of Dm domains of 12 Dmrt transcription factors—the colors correspond to different proteins; Figure S3: A heatmap of RMSD of DM domain of Dmrt transcription factors in *Scolopendra mutilans*.
