# Supplementary figures and images for: Comprehensive Genome-Wide Analysis of Dmrt Transcription Factors Reveals Their Roles in Sexual Dimorphism in Scolopendra mutilans
Source: Biology (Basel). 2025 Oct 20;14(10):1451. doi: 10.3390/biology14101451 (PMC12562212; doi:10.3390/biology14101451)

Figure S1

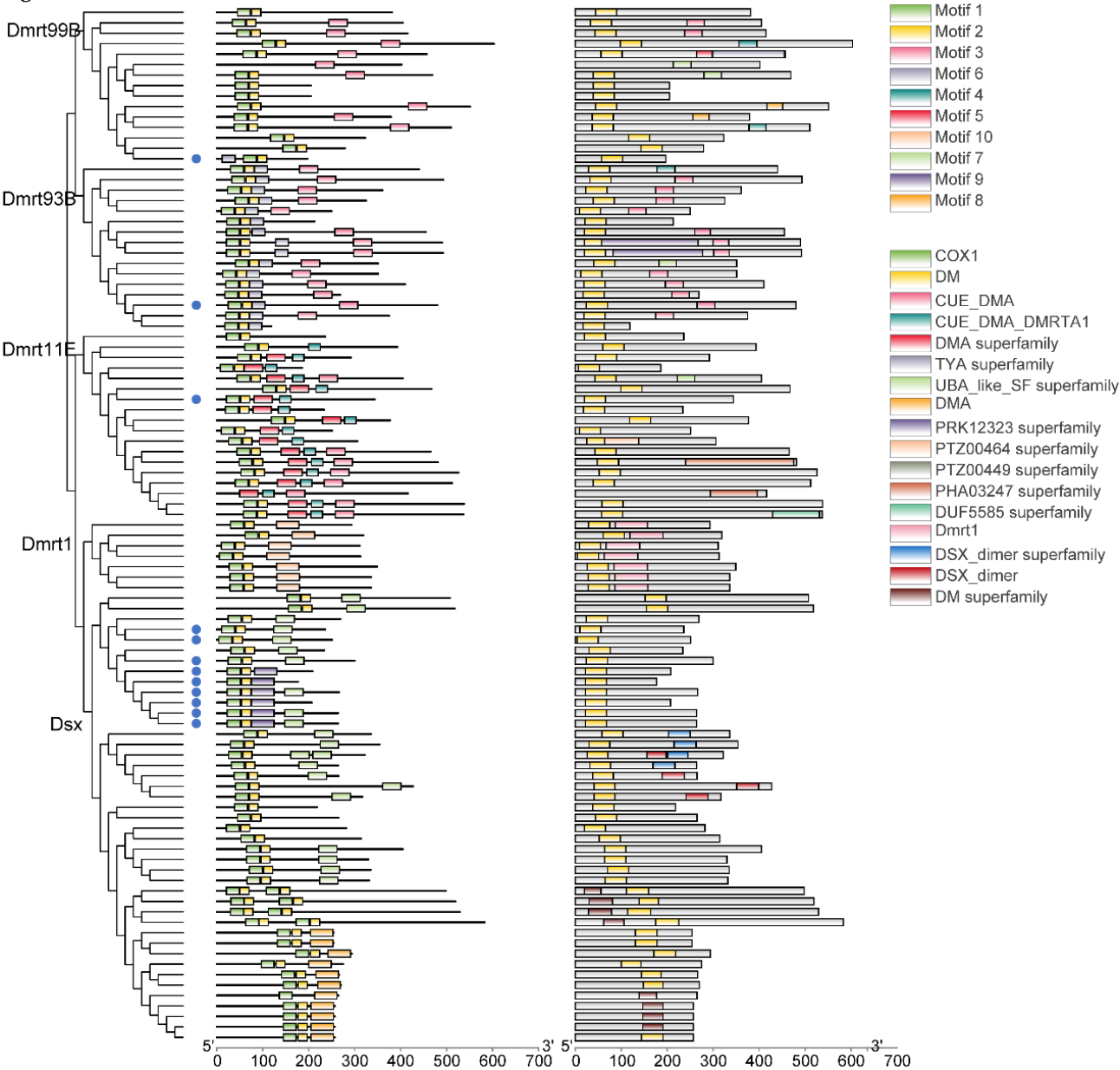

Figure S2

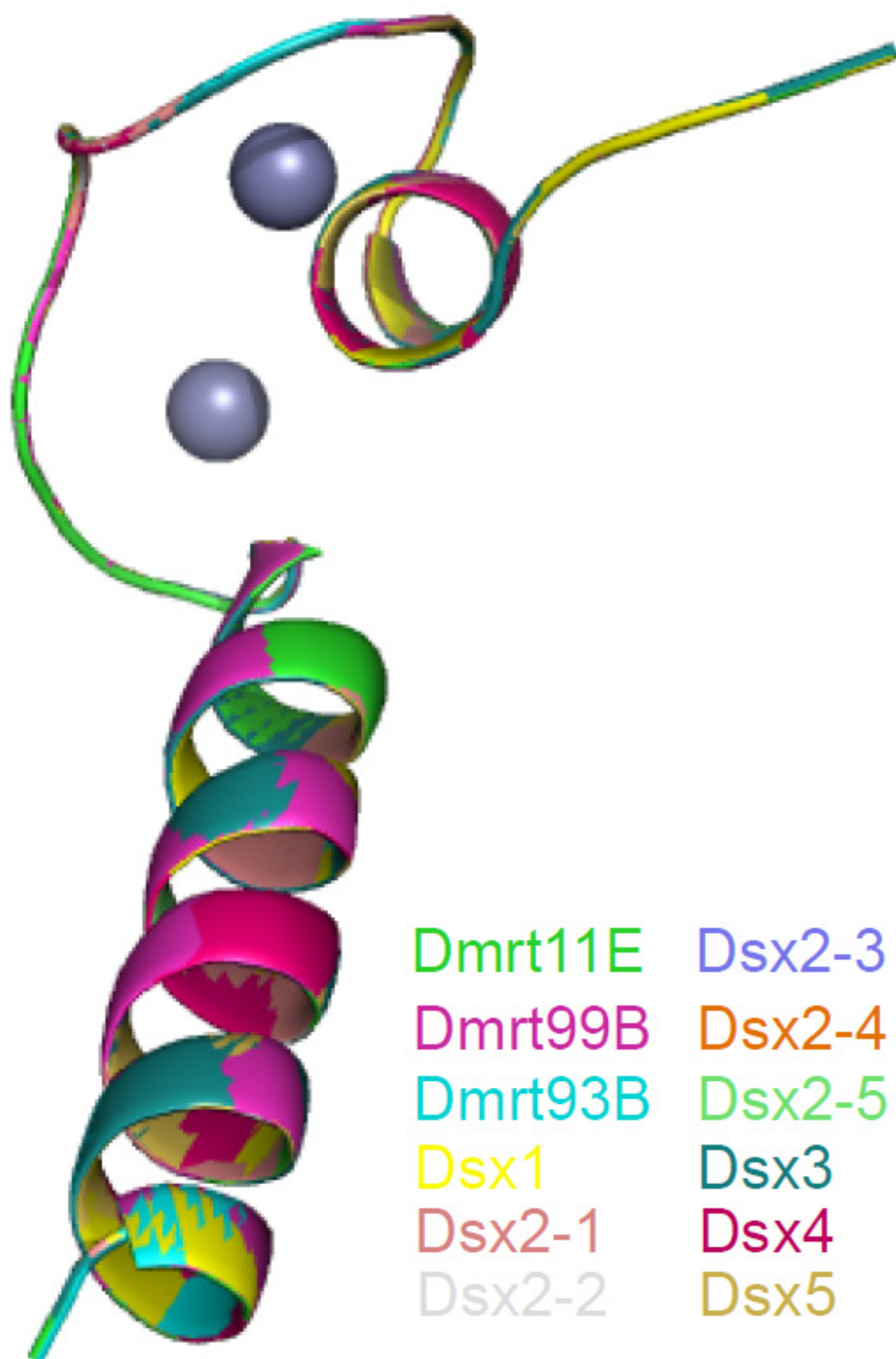

Figure S3

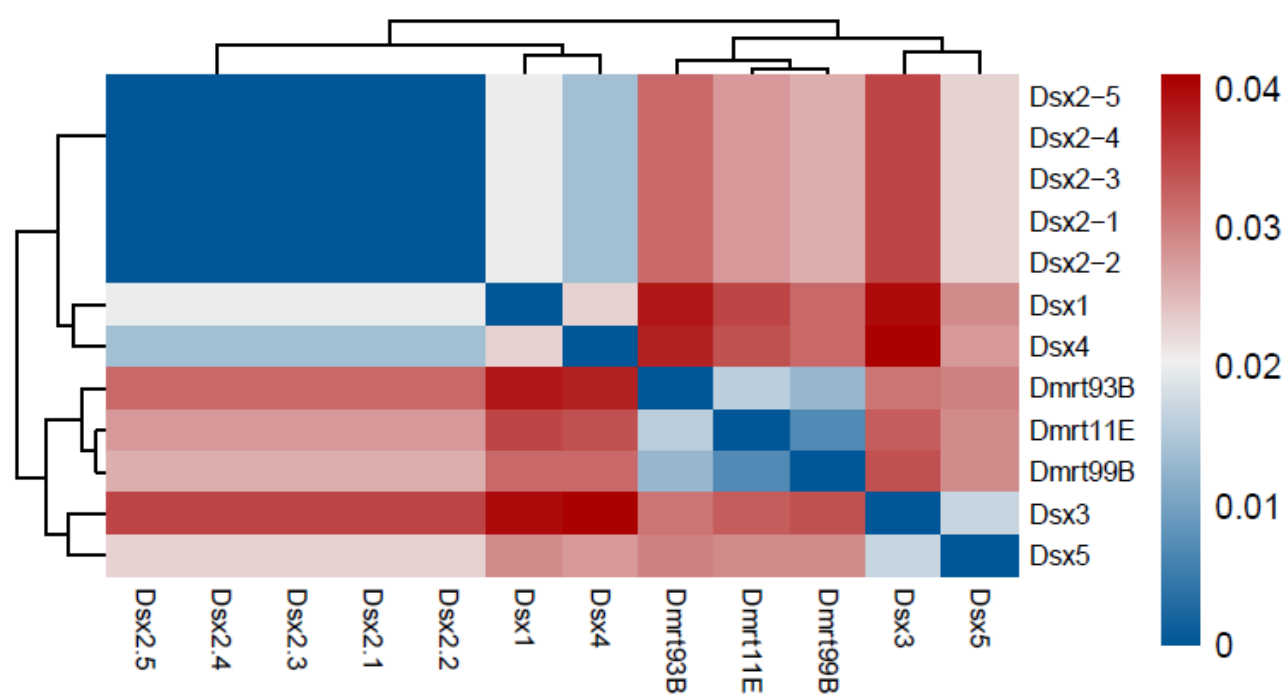

Supplement: Supplementary file 1 [file biology-14-01451-s001.zip › biology-3919457-supplementary.pdf]
